# Seasonal Alterations in Host Range and Fidelity in the Polyphagous Mirid Bug, *Apolygus lucorum* (Heteroptera: Miridae)

**DOI:** 10.1371/journal.pone.0117153

**Published:** 2015-02-18

**Authors:** Hongsheng Pan, Bing Liu, Yanhui Lu, Kris A. G. Wyckhuys

**Affiliations:** 1 State Key Laboratory for Biology of Plant Diseases and Insect Pests, Institute of Plant Protection, Chinese Academy of Agricultural Sciences, Beijing, China; 2 International Center for Tropical Agriculture CIAT-Asia, Hanoi, Vietnam; Centro de Investigación y de Estudios Avanzados, MEXICO

## Abstract

In herbivorous insects, host plant switching is commonly observed and plays an important role in their annual life cycle. However, much remains to be learned about seasonal host switching of various pestiferous arthropods under natural conditions. From 2006 until 2012, we assessed *Apolygus lucorum* (Meyer-Dür) host plant use in successive spring, summer and winter seasons at one single location (Langfang, China). Data were used to quantify changes in host plant breadth and host fidelity between seasons. Host fidelity of *A. lucorum* differed between seasons, with 87.9% of spring hosts also used in the summer and 36.1% of summer hosts used in winter. In contrast, as little as 25.6% host plant species were shared between winter and spring. Annual herbaceous plants are most often used for overwintering, while perennial woody plants are relatively important for initial population build-up in the spring. Our study contributes to an improved understanding of evolutionary interactions between *A. lucorum* and its host plants and lays the groundwork for the design of population management strategies for this important pest in myriad crops.

## Introduction

Due to temporal and spatial variation in host plant availability, and specific nutrition and/or habitat requirements of insects, most phytophagous insects exploit multiple plant species [1]. Also, many herbivores exhibit notable temporal variation in host plant use [2,3,4]. For example, the cherry-oat aphid, *Rhopalosiphum padi* (L.) and the cotton aphid, *Aphis gossypii* Glover use woody host plants as primary hosts, and switch to herbaceous plants as secondary hosts in the summer [5]. Host switching can be even more pronounced, with the southern green stink bug *Nezara viridula* L. adults frequently moving from one plant species to another [6]. Hence, the life history of such herbivores is inherently tied to their seasonal pattern of host switching [7,8].

As plant species temporally and spatially differ in nutritional quality, host plant switching can remediate qualitative nutritional impediments associated with certain host species [9] and help meet the differing nutritional requirements of certain developmental stages of a particular insect [10]. This behavioral compensation has been demonstrated in many herbivorous insects [11]. Moreover, some insect species prefer certain allelochemicals, nutritional profiles or physical characteristics of host plants at a given point in time [12,13]. By meeting specific nutritional demands or ensuring intake of certain phytochemicals, host plant switching can promote the survival and fecundity of multiple herbivorous insects [6,14,15,16].

In their exploitation of host plants, specialist herbivores exhibit high host fidelity (i.e., the tendency of a herbivore to use the same host plant species over time), whereas generalists show low fidelity [17]. Also, over the course of successive seasons, specialists only partially incorporate new plant species in their host repertoire and primarily use taxonomically, ecologically, or chemically similar plants [18,19,20,21]. The degree of host fidelity is often tied to feeding habits of the herbivorous insects, with internal feeders (e.g., miners, borers) exhibiting high host fidelity, whereas leaf feeders more likely to expand their host range [22]. Notwithstanding intensive host switching, generalists often exhibit clear preference for certain plant species, plant families or plant growth forms [1,23]. Despite an increasing amount of research on this topic, much remains unknown on host plant use and host switching in some common herbivores.

Mirid bugs (Hemiptera: Miridae) are found in natural and agricultural ecosystems throughout the world, and many of them are generalists, exhibiting diverse feeding habits or preferences (e.g., feeding on leaf, stem, inflorescences, nectar, pollen and fruit) [24]. Several mirid bugs, such as *Lygus lineolaris* (Palisot de Beauvois), frequently alter host plants and habitats to locate suitable food sources [25,26,27]. In China, the mirid bug *Apolygus lucorum* (Meyer-Dür) has become the primary pest of several key agricultural crops and fruit trees over the past decade [28,29]. This species overwinters as eggs in plant tissues from October to April. Subsequently, with five generations per year, *A*. *lucorum* adults exploit a broad range of host plants, including crops, fruit trees and common weed species [30,31]. To date, *A*. *lucorum* has been recorded on a total of 66 and 86 plant species during respective spring and winter seasons, and their host species greatly differed [32,33]. Hence, we further determined the host plant range of *A*. *lucorum* in summer season. We hypothesized that polyphagous *A*. *lucorum* would exhibit host switching and have low host fidelity between seasons. However, the degree to which *A*. *lucorum* relies on certain plant species, families or growth forms (inc. herbaceous and woody plants) during successive seasons remains to be quantified.

In this study, we analyzed multi-year data on *A*. *lucorum* host plant use [32,33]. Our research set out to detect seasonal patterns in *A*. *lucorum* exploitation of certain plant species, families or growth forms, and determine the degree of host fidelity and temporal changes in host breadth of this mirid bug. These findings will provide further insights into seasonal host plant use of polyphagous herbivores and contribute to the design of area-wide management tactics for this key pest in Chinese agro-landscapes [34,35,36].

## Materials and Methods

### Ethics statement

No specific permits were required for the described field studies.

### Field survey

Field surveys of *A*. *lucorum* host plant use were carried out from December to May 2006–2009 for winter hosts [32], from April to June 2006–2010 for spring hosts [33], and from July to September 2006–2012 for summer hosts. In order to standardize the sampling protocol across season, all assays were conducted at natural areas and agricultural fields (covering >500 ha.) around the Langfang Experiment Station of the Chinese Academy of Agricultural Sciences (CAAS) (116.4 °E, 39.3 °N), in Hebei Province, China. Each year, we sampled a broad range of common and widely-distributed plant species in study area, including agricultural crops, fruit and timber trees, pastures and weeds. Plant species were identified, using regional weed guides [34] or with the assistance of CAAS plant taxonomists. During each survey, the status (dead or alive) of all plant species was recorded. Plant species on which *A*. *lucorum* adults or nymphs were found were defined as ‘host plants’ of this species [27].

For the survey of winter hosts, above-ground parts of different plant species were collected in late March and subsequently placed in a 3 m^2^ screen-cage with about 10 horsebean plants (*Vicia faba* L.). Horsebean is an important host plant and suitable food source of *A*. *lucorum* [30,31]. Within each cage, we placed 1 m^2^ plant material of one plant species; and each plant species had 1–5 cages per year, depending on the amount of plant material. From April to May, when *A*. *lucorum* over-wintering eggs hatch and emergent nymphs move to surrounding horsebean plants for feeding, we surveyed nymphal density on the plants within each cage, using a standard white pan beating method [38]. Emergent *A*. *lucorum* nymphs indirectly indicated presence of its overwintering eggs, which are too small to be counted with the naked eye [32].

For the survey of spring host plants, we examined different plant species using a standard white pan beating method from mid-April to mid-June. More specifically, entire specimens of most herbaceous plants or young branches of fruit and other trees were immediately shaken over a 40 x 26 x 11 cm white pan, and the number of dislodged *A*. *lucorum* individuals in the pan was recorded [33,38]. To unequivocally record associations of *A*. *lucorum* with a given plant species, we only selected uniform patches or carefully picked single stems of a given plant species for sampling. Surveys were conducted every 3–5 days, and *A*. *lucorum* individuals were counted for each sample. As part of each sampling activity, the exact area covered by each sampled plant was recorded. For common plant species, 10–20 samples were randomly selected per survey date, and a single sample consisted of a total area of 2–5 m^2^, while for uncommon species, all plants at a given site were sampled. Sampling was repeated 11–16 times per year [33].

For the survey of summer host plants, we used the above method to sample *A*. *lucorum* from July to September. A total of 3 sampling events (once per month) were conducted per year, with 10–20 random samples taken per plant species and event. During the successive seven summers, a total of 252 species of plants were sampled and each plant species was sampled for at least 2 years.

### Statistical analysis

The average density of *A*. *lucorum* per plant species during each season was computed on a yearly basis, i.e., by dividing the total number of individuals on a given plant species by the total sample area covered by this respective plant. *A*. *lucorum* density was compared between different plant species using a two-way un-replicated ANOVA with year and plant species as fixed factors, if the data met normality and homogeneity of variance; otherwise, the data were analyzed using a non-parametric test (Friedman’s test) instead. Its average density per plant group (i.e., host type, plant growth form) during each season was also computed on a yearly basis. The density of *A*. *lucorum* was compared between different plant groups using a one-way ANOVA with a Tukey test. A Chi-square test was performed to compare between-season host use ratio (i.e., the proportion of host plants in one given season that is also used as food plants in the subsequent season). All statistical analyses were performed using SAS [39].

## Results

### Seasonal host breadth

**Fig 1:**
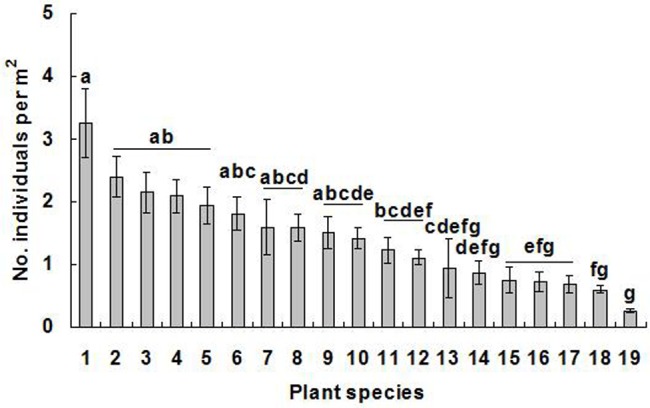
Population density of *Apolygus lucorum* on 18 preferred host plant species in the summers from 2006–2012 at Langfang (Hebei Province, Northern China). The average densities of *A*. *lucorum* on the 18 plant species were higher than 0.5 individuals per m^2^. The data in the figures were shown as mean ± SE. In each figure, bars with the same letters are not significantly different at the 0.05 level. Plant species: 1 *Artemisia lavandulaefolia* DC., 2 *Artemisia argyi* Lévl. et Vant., 3 *Vigna radiata* (L.) Wilczek, 4 *Artemisia annua* L., 5 *Artemisia scoparia* Waldst. et Kit., 6 *Impatiens balsamina* L., 7 *Agastache rugosus* (Fisch. et Meyer) O. kuntze., 8 *Cannabis sativa* L., 9 *Ricinus communis* L., 10 *Humulus scandens* (Lour.) Merr., 11 *Ocimum basilicum* L., 12 *Gossypium hirsutum* L., 13 *Amaranthus hypochondriacus* L., 14 *Polygonum orientale* L., 15 *Helianthus annuus* L., 16 *Mentha haplocalyx* Briq., 17 *Medicago sativa* L., 18 *Fagopyrum esculentum* Moench. The last bar (No. 19) showed that the mean density of *A*. *lucorum* on all tested plant species during these 7 years.

In summer, a total of 233 plant species from 49 families, including 61 agricultural crops, 19 trees, 13 pasture species, 64 cultivated (herbaceous) plants and 76 herbaceous weeds, were identified as host plants of *A*. *lucorum* (Table 1). Most important plant families were Asteraceae (40 species, accounting for 17.2% of the total), Fabaceae (30 species, 12.9%), Lamiaceae and Brassicaceae (13 species and 5.6% each), Rosaceae (11 species, 4.7%), and Poaceae and Solanaceae (10 species and 4.3% each). During the whole study period, no individual of *A*. *lucorum* was found on 19 plant species, including *Amtirrhinum majus* L., *Anemarrhena asphodeloides* Bunge, *Atractylodes macrocephala* Koidz., *Carex rigescens* (Franch.) V. Krecz, *Cicer arietium* L., *Codonopsis pilosula* (Franch.) Nannf., *Echinochloa crusgalli* (L.) Beauv., *Eleusine indica* (L.) Gaertn., *Equisctum ramosissimum* Desf., *Ginkgo biloba* L., *Liquidambar formosana* Hance, *Pinellia pedatisecta* Schott, *Platycladus orientalis* (L.) Franco, *Polygala tenuifolia* Willd., *Setaria viridis* (L.) Beauv., *Syringa vulgaris* L., *Trigonella foenum-graecum* L., *Vaccaria segetalis* (Necr.) Gracke., and *Zanthoxylum bungeanum* Maxim (Table 1). Throughout the study, average *A*. *lucorum* density on all host plant species was 0.27 ± 0.03 individuals per m^2^. On 18 host species, *A*. *lucorum* density proved higher than 0.50 individuals per m^2^. Population densities of *A*. *lucorum* significantly differed between plant species (*X* ^*2*^ = 86.09, df = 18, *P* < 0.0001) (Fig. 1).

**Table 1:** Host plant species of *Apolygus lucorum* in the summer and other two seasons at Langfang (Hebei Province, Northern China).

**Family**	**Plant species**	**Plant growth forms**	**Summer hosts**	**winter hosts**	**Spring hosts**
Amaranthaceae	*Achyranthes bidentata* Blume	O	√	√	
	*Amaranthus caudatus* L.	W	√		
	*Amaranthus hypochondriacus* L.	P	√	√	
	*Amaranthus retroflexus* L.	W	√	√	√
	*Amaranthus tricolor* L.	V	√	√	
	*Celosia cristata* L.	O	√	√	
	*Gomphrena globosa* L.	O	√	×	
Apocynaceae	*Catharanthus roseus* (L.) G. Don	O	√		
Araceae	*Arisaema erubescens* (Wall.) Schott	O	√		
	*Pinellia pedatisecta* Schott	O	×		
Asclepiadaceae	*Cynanchum chinense* R. Br.	W	√		√
	*Cynanchum thesioides* (Freyn) K. Schum.	W	√		×
	*Metaplexis japonica* (Thunb.) Mak.	W	√		×
	*Telosma cordata* (Burm. f.) Merr.	O	√		
Asteraceae	*Achillea millefolium* L.	W	√		
	*Ageratum conyzoides* L.	W	√	√	
	*Arctium lappa* L.	O	√	×	
	*Artemisia annua* L.	W	√	√	√
	*Artemisia argyi* Lévl. et Vant．	W	√	√	√
	*Artemisia lavandulaefolia* DC.	W	√	√	√
	*Artemisia scoparia* Waldst. et Kit.	W	√	√	√
	*Atractylodes macrocephala* Koidz.	W	×		
	*Bidens pilosa* L.	W	√		×
	*Calendula officinalis* L.	O	√		
	*Carduus crispus* L.	W	√		√
	*Carthamus tinctorius* L.	O	√	√	
	*Cephalanoplos setosum* (Willd.) Kitam.	W	√		√
	*Chamaemelum nobile* (L.) All.	O	√		
	*Chrysanthemum coronarium* L.	V	√	√	
	*Chrysanthemum paludosum* L.	O	√		
	*Cichorium intybus* L.	P	√	×	
	*Cirsium japonicum* DC.	W	√		
	*Cirsium setosum* (Willd.) MB.	W	√		√
	*Conyza canadensis* (L.) Cronq．	W	√		√
	*Coreopsis basalis* L.	O	√		
	*Coreopsis tinctoria* Nutt.	O	√		
	*Cosmos sulphureus* Cav.	O	√		
	*Dendranthema morifolium* (Ramat.) Tzvel.	O		×	
	*Erigeron annuus* (L.) Pers.	W	√		
	*Helianthus annuus* L.	A	√	×	
	*Hemistepta lyrata* Bunge	W	√		√
	*Heteropappus altaicus* (Willd.) Novopokr.	W	√		√
	*Inula japonica* Thunb.	W	√		×
	*Ixeris chinensis* (Thunb.) Nakai.	W			√
	*Ixeris denticulata* (Houtt.) Stebb.	W	√	√	
	*Ixeris sonchifolia* Hance	W	√		
	*Lactuca indica* L.	W	√	√	√
	*Lactuca sativa* L.	V	√	×	
	*Pyrethrum cinerariifolium* Trev.	O	√	×	
	*Rudbeckia hirta* L.	O	√		
	*Sonchus brachyotus* DC.	W	√		
	*Sonchus oleraceus* L.	W			×
	*Tagetes eracta* L.	O	√		
	*Tagetes patula* L.	O	√		
	*Taraxacum brassicaefolium* Kitag.	W	√		
	*Taraxacum mongolicum* Hand.-Mazz.	W	√	×	√
	*Xanthium sibiricum* Patrin ex Widder	W	√	√	√
	*Zinnia elegans* Jacq.	O	√	×	
Balsaminaceae	*Impatiens balsamina* L.	O	√	√	
Basellaceae	*Basella rubra* L.	V	√	×	
Begoniaceae	*Begonia grandis* Dry.	F			×
	*Borago officinalis* L.	O	√		
	*Bothriospermum chinense* Bge.	W			√
	*Echium vulgare* L.	O	√		
	*Lithospermum erythrorhizon* Sieb. et Zucc.	W	√		
	*Lycopsis orientalis* L.	W	√		√
Brassicaceae	*Brassica albograbra* L. H. Bailey	V	√	√	
	*Brassica campestris* L.	V	√		
	*Brassica chinensis* L.	V	√		
	*Brassica juncea* (L.) Czern. et Coss.	V	√	√	
	*Brassica oleracea* L.	V	√	√	
	*Brassica pekinensis* Rupr.	V	√		
	*Capsella bursa-pastoris* (L.) Medic.	W			√
	*Descurainia Sophia* (L.) Webb. ex Prantl	W			√
	*Iberis amara* L.	O	√		
	*Isatis indigotica* Fort.	O	√	√	
	*Lepidium apetalum* Willd.	W	√		√
	*Orychophrapmus violaceus* (L.) O. E. Schulz	O	√		
	*Raphanus sativus* L.	V	√	√	
	*Rorippa islandica* (Oed.) Borb.	W	√		×
	*Sinapis alba* L.	V	√		
Campanulaceae	*Codonopsis pilosula* (Franch.) Nannf.	O	×		
	*Platycodon grandiflorus* (Jacq.) A. DC.	O	√	×	
Capparaceae	*Cleome gynandra* L.	O	√	√	
	*Cleome spinosa* Jacq.	O	√		
Caryophyllaceae	*Dianthus superbus* L.	O	√	√	
	*Vaccaria segetalis* (Necr.) Gracke.	O	×		
Chenopodiaceae	*Beta vulgaris* L.	A	√	×	
	*Chenopodium album* L.	W	√	√	√
	*Chenopodium glaucum* L．	W	√		√
	*Chenopodium serotinum* L.	W	√		√
	*Chenopodium urbicum* L.	W	√		
	*Kochia scoparia* (L.) Schrad.	W	√	√	√
	*Salsola collina* Pall.	W	√	√	√
	*Spinacia oleracea* L.	V	√		
	*Suaeda glauca* Bunge	W	√		
Convolvulaceae	*Calystegia dahurica* (Herb.) Choisy	W	√		
	*Calystegia hederacea* Wall.	W	√		√
	*Convolvulus arvensis* L.	W	√		√
	*Convolvulus tricolor* L.	W	√		
	*Ipomoea aquatica* Forsk.	V	√	×	
	*Ipomoea batatas* Lam.	A	√		
	*Pharbitis nil* (L.) Choisy	O	√	√	
	*Pharbitis purpurea* (L.) Voight	W	√		
Cucurbitaceae	*Benincasa hispida* (Thunb.) Cogn.	V	√	√	
	*Citrullus lanatus* (Thunb.) Mansfeld	V	√		
	*Cucumis melo* L.	V	√		
	*Cucumis sativus* L.	V	√		
	*Cucurbita moschata* (Duch.) Poiret	V	√	√	
	*Cucurbita pepo* L.	V	√	×	
	*Luffa cylindrica* (L.) Roem.	V	√	×	
	*Momordica charantia* L.	V	√	√	
	*Trichosanthes kirilowii* Maxim.	O	√	×	
Cupressaceae	*Platycladus orientalis* (L.) Franco	E	×		×
Cyperaceae	*Carex rigescens* (Franch.) V. Krecz	W	×		×
Dioscoreaceae	*Dioscorea opposita* Thunb.	A	√		
Ebenaceae	*Diospyros kaki* L.	F	√		√
Equisetaceae	*Equisctum ramosissimum* Desf.	W	×		×
Euphorbiaceae	*Acalypha australis* L.	W	√		
	*Euphorbia esula* L.	W	√		√
	*Euphorbia humifusa* Willd.	W	√		
	*Euphorbia marginata* Pursh.	O	√		
	*Ricinus communis* L．	A	√	√	
Fabaceae	*Amorpha fruticosa* L.	E	√	×	√
	*Arachis hypogaea* L.	A	√	×	
	*Astragalus adsurgens* Pall.	P	√		
	*Astragalus complanatus* Bunge	O	√	√	
	*Cassia occidentalis* L.	O	√	√	
	*Cassia tora* L.	O	√	√	
	*Cicer arietium* L.	A	×		
	*Coronilla varia* L.	P	√	√	√
	*Dolichos lablab* L.	O	√	√	
	*Glycine max* (L.) Merr.	A	√	√	
	*Glycyrrhiza uralensis* Fisch.	O	√	√	
	*Gueldenstaedtia multiflora* Bunge	W			×
	*Lablab purpureus* (L.) Sweet	V	√	√	
	*Medicago falcata* L.	P	√		
	*Medicago sativa* L.	P	√	√	√
	*Melilotus albus* Desr.	P	√	√	
	*Melilotus suaveolens* Ledeb.	P	√		√
	*Mimosa pudica* L.	W	√		
	*Onobrychis viciifolia* Scop.	P	√	√	√
	*Phaseolus coccineus* L.	V	√		
	*Phaseolus vulgaris* L.	V	√	√	
	*Pisum sativum* L.	V	√		
	*Robinia pseudoacacia* L.	E	√		√
	*Sophora flavescens* Ait.	W	√		
	*Sophora japonica* L.	E	√		
	*Trifolium pratense* L.	P	√	√	
	*Trifolium repens* L.	P	√	×	
	*Trigonella foenum-graecum* L.	O	×		
	*Vicia faba* L.	A		√	√
	*Vicia villosa* Roth	P	√	√	
	*Vigna angularis* (Willd.) Ohwi et Ohashi	A	√	√	
	*Vigna radiata* (L.) Wilczek	A	√	√	
	*Vigna umbellata* (Thunb.) Ohwi et Ohashi	A	√		
	*Vigna unguiculata* (L.) Walp.	V	√	√	
Ginkgoaceae	*Ginkgo biloba* L.	E	×		×
Hamamelidaceae	*Liquidambar formosana* Hance	E	×		×
Juglandaceae	*Juglans regia* L.	E			×
Lamiaceae	*Agastache rugosus* (Fisch. et Meyer) O. kuntze.	O	√	√	
	*Hyssopus officinalis* L.	W	√		
	*Lagopsis supina* (Steph.) IK.-Gal.	W			√
	*Leonurus heterophyllus* Sweet	W	√	√	
	*Leonurus sibiricus* L.	W	√		√
	*Marjoraan hortensis* Moenoh. syn. Origanum	O	√	√	
	*Mentha haplocalyx* Briq.	A	√	√	
	*Ocimum basilicum* L.	O	√	√	
	*Salvia farinacea* Benth.	O	√	×	
	*Salvia officinalis* L.	O	√	√	
	*Salvia plebeia* R. Br.	W	√		√
	*Salvia splendens* Ker-Gawler	O	√	√	
	*Schizonepeta tenuifolia* (Benth.) Briq.	O	√	√	
	*Scutellaria baicalensis* Georgi	O	√	√	
Liliaceae	*Allium fistulosum* L.	V	√	×	×
	*Allium tuberosum* Rottl. ex Spreng.	V	√	×	
	*Anemarrhena asphodeloides* Bunge	O	×	×	
Linaceae	*Linum usitatissimum* L.	A	√	×	
Malvaceae	*Abelmoschus esculentus* (L.) Moench.	V	√	×	
	*Abutilon theophrasti* Medic.	W	√	√	√
	*Althaea rosea* (L.) Cavan.	O	√		
	*Gossypium hirsutum* L.	A	√	√	
	*Hibiscus cannabinus* L.	A	√	√	
	*Malope trifida* L.	O	√		
	*Malva sinensis* Cavan.	O	√		
Moraceae	*Cannabis sativa* L.	A	√	√	√
	*Humulus scandens* (Lour.) Merr.	W	√	√	√
	*Morus alba* L.	E	√		√
Nyctaginaceae	*Mirabilis jalapa* L.	O	√		
Oleaceae	*Forsythia suspensa* (Thunb.) Vahl	O	√	×	
	*Syringa vulgaris* L.	E	×		×
Onagraceae	*Oenothera odorata* Jacq.	O	√		
Oxalidaceae	*Oxalis corniculata* L.	W	√		×
Pedaliaceae	*Sesamum indicum* L.	A	√	√	
Phytolaccaeae	*Phytolacca acinosa* Roxb.	W	√	√	
Plantaginaceae	*Plantago asiatica* L.	W	√		
	*Plantago depressa* Willd.	W	√		√
Poaceae	*Alopecurus japonicus* Steud.	W			√
	*Coix lacryma-jobi* L.	O	√	√	
	*Echinochloa crusgalli* (L.) Beauv.	W	×		×
	*Eleusine indica* (L.) Gaertn.	W	×		×
	*Hordeum vulgare* L.	A	√		
	*Imperata cylindrica* (L.) Beauv.	W	√		√
	*Leptochloa chinensis* (L.) Nees.	O	√	√	
	*Oryza sativa* L.	A	√		
	*Phragmites communis* Trin.	W	√		√
	*Poa annua* L．	W			×
	*Setaria italica* (L.) Beauv.	A	√	√	
	*Setaria viridis* (L.) Beauv.	W	×	×	×
	*Sorghum sudanense* (Piper) Stapf	P	√		
	*Sorghum vulgare* Pers.	A	√	√	
	*Triticum aestivum* L.	A			√
	*Zea mays* L.	A	√	√	
Polemoniaceae	*Phlox drummondii* Hook.	O	√		
Polygonaceae	*Fagopyrum esculentum* Moench	A	√	√	
	*Polygala tenuifolia* Willd.	O	×	×	
	*Polygonum aviculare* L.	W	√		√
	*Polygonum lapathifolium* L.	W			×
	*Polygonum orientale* L.	W	√	×	
	*Rheum officinale* Baill.	W	√		
	*Rumex acetosa* L.	W	√		
	*Rumex dentatus* L.	W			×
Portulacaceae	*Portulaca grandiflora* Hook.	O	√		
	*Portulaca oleracea* L.	W	√		√
Ranunculaceae	*Nigella damascena* L.	O	√		
Rhamnaceae	*Ziziphus jujuba* Mill.	F	√	√	√
Rosaceae	*Cerasus pseudocerasus* (Lindl.) G. Don	F	√		√
	*Crataegus pinnatifida* Bge.	F	√		√
	*Fragaria ananassa* Duchesne	F	√		
	*Malus prunifolia* (Willd.) Borkh.	F	√	√	
	*Malus pumila* Mill.	F	√	√	√
	*Potentilla chinensis* Ser.	W	√		
	*Potentilla supina* L.	W	√		√
	*Prunus armeniaca* L.	F	√	×	√
	*Prunus cerasifera* Ehrh.	F		√	
	*Prunus persica* L.	F	√	√	√
	*Prunus salicina* Lindl.	F	√		√
	*Pyrus bretschneideri* Rehd.	F	√	√	√
Rubiaceae	*Ixora chinensis* Lam.	W	√		
	*Rubia cordifolia* L.	W	√		√
Rutaceae	*Murraya paniculat* (L.) Jack.	O	√		
	*Zanthoxylum bungeanum* Maxim.	E	×		×
Salicaceae	*Populus tomentosa* Carr.	E		×	×
	*Salix matsudana* Koidz.	E	√	×	√
Scrophulariaceae	*Lindernia procumbens* (Krock.) Philcox	W	√		×
	*Rehmannia glutinosa* Libosch.	W	√		√
	*Amtirrhinum majus* L.	W	×		
Simaroubaceae	*Ailanthus altissima* Swingle	E	√		√
Solanaceae	*Capsicum annuum* L.	V	√	×	
	*Datura metel* L.	O	√		
	*Datura stramonium* L.	O	√	×	
	*Lycopersicon esculentum* Mill.	V	√	×	
	*Nicotiana tabacum* L.	O	√		
	*Petunia hybrida* Vilm.	O	√		
	*Physalis alkekengi* L.	W	√	√	
	*Solanum melongena* L.	V	√	√	
	*Solanum nigrum* L．	W	√	×	
	*Solanum tuberosum* L.	V	√	×	
Tiliaceae	*Corchorus capsularis* L.	A	√	√	
Ulmaceae	*Ulmus pumila* L.	E	√	×	√
Umbelliferae	*Angelica dahurica* (Fisch. ex Hoffm.) Benth. et Hook. f.	O	√	√	
	*Apium graveolens* L.	V	√	×	
	*Bupleurum falcatum* L．	O	√	√	
	*Cnidium monnieri* (L.) Cuss.	W	√		×
	*Coriandrum sativum* L.	V	√	√	
	*Daucus carota* L.	V	√	√	
	*Daucus carota* var. sativa DC.	V	√		
	*Saposhnikovia divaricata* (Turcz.) Schischk.	O	√	×	
Violaceae	*Viola prionantha* Bunge	W			×
Vitaceae	*Vitis vinifera* L.	F	√	√	√
Zygophyllaceae	*Tribulus terrester* L.	W	√	√	

Note: Information on winter and spring host ranges is cited from documents [32,33]. The signs “√” and “×” indicate that the according plant species is a host plant and non-host plant, respectively. A blank in this column indicates no assay. A = Agricultural crops, E = Economic trees, F = Fruit trees, O = Other cultivated plants (except for A, E, F, P, V and W), P = Pasture crops, V = Vegetable crops, W = Weeds.

In the winter, *A*. *lucorum* eggs successfully hatched on 86 plant species, among which 16 weeds and fruit trees were considered as key overwintering hosts [32].

In the spring, 66 plant species were found to be hosts of *A*. *lucorum*. Among these species, 6 hosts were identified as dominant host species due to their wide distribution and high population densities of *A*. *lucorum* [33].

### Between-season host fidelity

**Fig 2:**
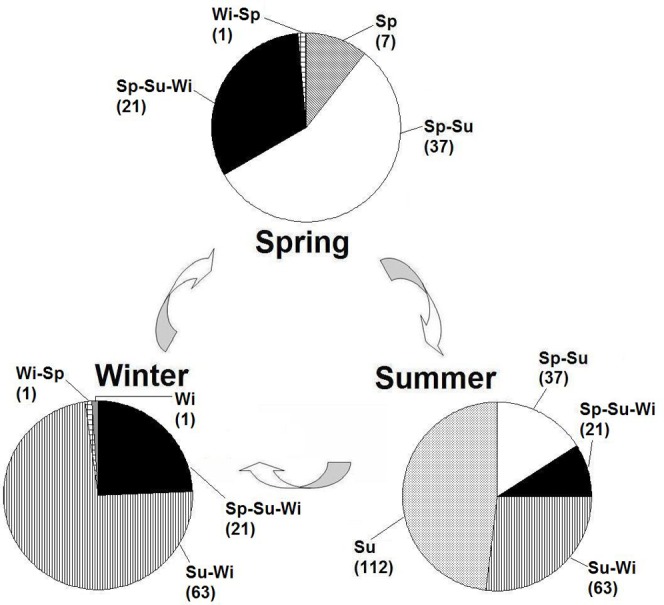
Seasonal host switching of *Apolygus lucorum* among different plant species at Langfang (Hebei Province, Northern China). Sp-Su-Wi refers to species of host plants through spring, summer and winter seasons. Sp-Su and Su-Wi are host plants in both spring and summer, and both summer and winter, respectively. Sp, Su and Wi are those only in spring, in summer and in winter, respectively. Data between brackets refer to the number of corresponding host plant species.

**Host plant species**. Over the course of a year, *A*. *lucorum* exhibited a highly variable host breadth, using 66 host plants in spring, 233 in summer and 86 in winter (Table 2). Of the spring host plants, 87.9% (58/66 species) were also used as summer hosts, while 36.1% (84/233 species) of summer hosts were used for overwintering. Only 25.6% (22/86 species) of winter hosts also served as spring hosts. Host use ratios significantly differed between successive seasons (*X* ^*2*^ = 69.61, df = 2, *P* < 0.0001) (Fig. 2).

**Table 2:** A summary of host plant use of *Apolygus lucorum* in the summer and other two seasons at Langfang (Hebei Province, Northern China).

Season	No. host plants	Host types							Plant growth forms		
		Sp	Su	Wi	Sp-Su	Su-Wi	Wi-Sp	Sp-Su-Wi	Ah	Ph	Pw
Spring	66	7	0	0	37	0	1	21	31	19	16
Summer	233	0	112	0	37	63	0	21	143	68	22
Winter	86	0	0	1	0	63	1	21	60	19	7
Total	242	7	112	1	37	63	1	21	149	70	23

Note: For family types, Sp-Su-Wi refers to families of host plants through spring, summer and winter seasons; Sp-Su, Su-Wi and Su are host plants in both spring and summer, in both summer and winter, and only in summer, respectively. For host types, Sp-Su-Wi refers to species of host plants through spring, summer and winter seasons; Sp-Su and Su-Wi are host plants in both spring and summer, and both summer and winter, respectively; Sp, Su and Wi are those only in spring, in summer and in winter, respectively. For plant growth forms, Ah, Ph and Pw are the annual herbaceous plants, perennial herbaceous plants, and perennial woody plants, respectively.

Natural plant death led to 58 (of 66) spring host plants being monitored during summer, and 134 (of 233) summer host plants monitored during the winter. Lastly, only 63 (of 86) winter hosts were alive during spring, of which 35 plant species were at seedling stage (Table 3).

**Table 3:** Survival of *Apolygus lucorum* host plants over subsequent seasons at the Langfang experiment station and surroundings (Hebei Province, Northern China).

Season (x)			Season (x+1)		
Season	Host type	No. plant species	Season	No. surviving plant species	Percent host plant survival (%)
Spring	Sp	7	Summer	0	0
	Sp-Su	37		37	100.00
	Sp-Su-Wi	21		21	100.00
	Wi-Sp	1		0	0
Summer	Sp-Su	37	Fall (Winter)	11	29.73
	Sp-Su-Wi	21		21	100.00
	Su-Wi	63		63	100.00
	Su	112		39	34.82
Winter (Fall)	Sp-Su-Wi	21	Spring	21	100.00
	Su-Wi	63		63 (35)	100.00 (55.56)
	Wi-Sp	1		1	100.00
	Wi	1		0	0

Note: Sp-Su-Wi refers to *A*. *lucorum* host plants during spring, summer and winter seasons. Sp-Su and Su-Wi are host plants in both spring and summer, and both summer and winter, respectively. Sp, Su and Wi are those only reported from spring, summer, or winter, respectively. Data within brackets show the number of plants at seedling stage, thus unable to support *A*. *lucorum* population development.

**Plant growth forms**. Among all different species of host plants, 149, 70 and 23 species were annual herbaceous plants, perennial herbaceous plants and perennial woody plants, respectively. The extent to which distinct plant growth forms were used as *A*. *lucorum* hosts was significantly different between successive seasons (herbaceous and woody plants: *X* ^*2*^ = 11.15, df = 2, *P* = 0.0038; annual and perennial plants: *X* ^*2*^ = 11.02, df = 2, *P* = 0.0041; three plant growth forms: *X* ^*2*^ = 14.58, df = 4, *P* = 0.0057). Annual herbaceous plants accounted for 47.0%, 61.4% and 69.8% of all host plants in spring, summer and winter, respectively; perennial herbaceous plants being 28.8%, 29.2% and 22.1%, and perennial woody plants occupying 24.2%, 9.4% and 8.1% in the distinct seasons (Table 4).

**Table 4:** Growth forms of plant species used as host plants by *Apolygus lucorum* during single and subsequent seasons.

Host type	Total no. plant species	Plant growth form		
		Annual herbaceous	Perennial herbaceous	Perennial woody
Sp	7	5/71.43%	2/28.57%	0/0
Su	112	69/61.61%	38/33.93%	5/4.46%
Wi	1	0/0	0/0	1/100.00%
Sp-Su	37	15/40.54%	11/29.73%	11/29.73%
Su-Wi	63	49/77.78%	13/20.63%	1/1.59%
Wi-Sp	1	1/100.00%	0/0	0/0
Sp-Su-Wi	21	10/47.62%	6/28.57%	5/23.81%
Total	242	149/61.57%	70/28.93%	23/9.50%

Note: The data above the diagonal line are the numbers of host species, and those below the diagonal line are the corresponding proportions in each host type.

**Population levels**. In spring, *A*. *lucorum* densities were significantly different between four categories of host plants, including Sp (host plants used only for spring), Sp-Su (for spring and summer), Sp-Su-Wi (for spring, summer and winter), and Wi-Sp (for winter and spring) (F = 12.20, df = 3,16, *P* = 0.0004). More specifically, seasonal density of *A*. *lucorum* on Wi-Sp during the spring was significantly higher than in the three other categories. In summer, its densities also significantly differed between the following plant categories: Sp-Su, Sp-Su-Wi, Su (only for summer) and Su-Wi (for summer and winter) (F = 37.80, df = 3,24, *P* < 0.0001). More so, *A*. *lucorum* density on Sp-Su-Wi and Su-Wi was significantly higher than on Sp-Su and Su. In winter, *A*. *lucorum* densities on Sp-Su-Wi, Su-Wi, Wi (only for winter) and Wi-Sp varied within the range of 0.3–1.6 individuals per square meter and did not differ between plant categories (F = 1.18, df = 2,6, *P* = 0.3698) (Table 5).

**Table 5:** Seasonal population densities of *Apolygus lucorum* on different groups of host plants during 2006–2012 at Langfang (Hebei Province, Northern China).

Season	Host type							Plant growth form		
	Sp	Sp-Su	Sp-Su-Wi	Su	Su-Wi	Wi	Wi-Sp	Annual herbaceous	Perennial herbaceous	Perennial woody
Spring	0.08 ± 0.05 (5) bc	0.05 ± 0.02 (5) c	0.23 ± 0.04 (5) b	-	-	-	0.48 ± 0.09 (2) a	0.09 ± 0.02 (5) a	0.24 ± 0.07 (5) a	0.14 ± 0.03 (5) a
Summer	-	0.04 ± 0.01 (7) b	0.41 ± 0.05 (7) a	0.07 ± 0.01 (7) b	0.35 ± 0.04 (7) a	-	-	0.26 ± 0.03 (7) b	0.41 ± 0.06 (7) a	0.05 ± 0.01 (7) c
Winter (fall)	-	-	1.62 ± 0.47 (3) a	-	0.96 ± 0.40 (3) a	0.33 (1)	0.58 ± 0.58 (3) a	0.94 ± 0.42 (3) a	0.99 ± 0.31 (3) a	1.15 ± 0.61 (3) a

Note: Differing letters indicate significant differences between one host type or plant growth form within one row (Tukey test, *P* < 0.05). Data within brackets show the number of sampling years (i.e., replications). The symbol “–” indicates no data. Data of *A*. *lucorum* density on each host plant in spring and winter seasons have been described earlier [32,33].

Among three different plant forms (e.g., annual herbaceous plants, perennial herbaceous plants and perennial woody plants), *A*. *lucorum* densities significantly differed in summer season (F = 25.42, df = 2,18, *P* < 0.0001), but not for spring (F = 2.47, df = 2,12, *P* = 0.1265) and winter (F = 0.05, df = 2,6, *P* = 0.9486). In summer, its densities on perennial herbaceous plants, annual herbaceous plants, and perennial woody plants significantly decreased in turn (Table 5).

**Fig 3:**
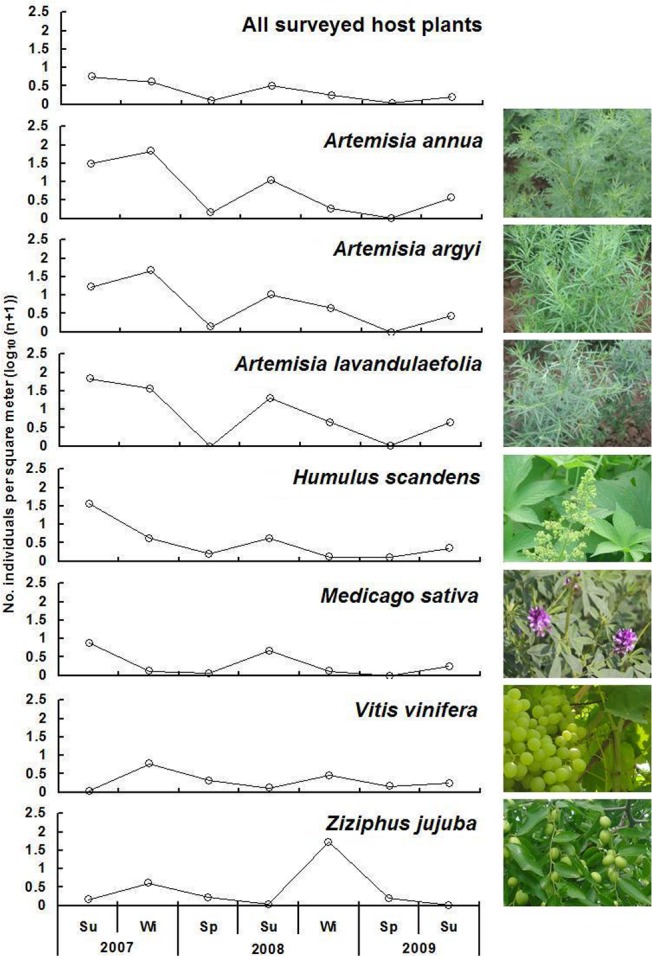
Seasonal population densities of *Apolygus lucorum* on the 7 principal host plants during 2007–2009 at Langfang (Hebei Province, Northern China). A total of 21 species were used as host plants throughout spring, summer and winter. Among 21 year-round hosts, only 7 species were found with relatively high *A*. *lucorum* population [32,33].

Over the course of a given year, *A*. *lucorum* overall density showed considerable variation, with the highest population levels recorded during summer and the lowest densities during winter. Among 21 year-round hosts, 7 species were found with relatively high *A*. *lucorum* population. On *Artemisia annua*, *Artemisia argyi* and *Artemisia lavandulaefolia*, *A*. *lucorum* attained high population densities in the summer and winter, and low density in the spring. Its population levels were high in the summer on *H*. *scandens* and *M*. *sativa*, whereas its density peaked in the winter on *Vitis vinifera* L. and *Ziziphus jujuba* Mill. (Fig. 3).

## Discussion

Host plant use and host switching play an important role in the evolutionary success of herbivorous insects [1,40,41]. To date, the host plant range of various mirid bugs has been determined, including *Lygus hesperus* Knight (110 species from 24 families) [42], *Lygus lineolaris* Palisot de Beauvois (385 species, 55 families) [43], *Lygus rugulipennis* Poppius (437 species, 57 families) [44], and *Pseudatomoscelis seriatus* (Reuter) (169 species, 35 families) [45]; and seasonal host switching has been documented for species, such as *L*. *lineolaris* [25,26,27], and *P*. *seriatus* [45]. For example, in Texas (USA), *Rapistrum rugosum* L. Allioni and *Ratibida columnifera* (Nuttall) Wooton and Standley were primary weed hosts of *L*. *lineolaris* during the early season, *Conyza canadensis* L. Cronquist and *Ambrosia trifida* L. were primary weed hosts during the midseason and late-season, respectively. *Sisymbrium irio* L. and *Lamium amplexicaule* L. sustained *L*. *lineolaris* populations during the overwintering period [27]. This past work further exemplifies the wide range of host plants and high frequency of host switching of certain species of herbivorous mirid bugs. In our study, we identified 233 species of summer host plants from 49 families for *A*. *lucorum*. Combining these data with literature records [31,32,33], we found *A*. *lucorum* to be associated with 288 plant species in 54 families and noted important seasonal changes in its host plant range. Although the possibility of individual transfer between different plant species was not fully evaluated and limited sampling might lead to underestimate its host range, the results from this 7-year study definitely provide a comprehensive set of information on host plant range of *A*. *lucorum*, especially for identifying the dominant ones. This pattern of multiple-host plant use of *A*. *lucorum* may be one of the reasons why *A*. *lucorum* quickly attained pest status in several agricultural crops throughout China [29].

Host plant diversity and food quality have been identified as the major drivers of host switching in herbivorous insects [41,46,47]. Our work shows host switching in *A*. *lucorum* to be likely determined by seasonal density of the insect and local availability of its host plants. Possibly, high *A*. *lucorum* density during summer months could explain its broader host breadth in this season [25]. Despite the fact that many new plant species are incorporated in *A*. *lucorum* diet over summer (i.e., 112 exclusive summer hosts and 63 hosts for both summer and winter), its spring hosts appear to be largely employed during summer. Also, as little as 12.1% of its spring hosts (i.e., 8 of 66) get abandoned in summer due to their absence during that season. Similar food requirements of *A*. *lucorum* populations and identical host plant phenology may further explain similarities in host plant use between these two successive seasons [31].

Host plant use showed certain differences between summer and fall/winter, with 149 summer plant species abandoned but only 2 new plant species incorporated in the *A*. *lucorum* winter host repertoire. Similar *A*. *lucorum* feeding requirements between summer and fall/winter may lead to high plant fidelity between seasons. However, especially for herbaceous annuals, plant senescence lead to 99 plant species being abandoned as hosts (Table 3).

On the other hand, host plant use between winter and spring greatly differs, with only 25.6% of winter hosts used in spring. We suspect that winter hosts mainly act as refuge and (over-winter) oviposition sites for *A*. *lucorum*, while spring hosts are primarily selected as food sources for hatched nymphs [31]. The distinct ecological function of winter and spring hosts might explain low host plant fidelity between these seasons. Also, our results suggest that *A*. *lucorum* adults select winter plants mainly for their food quality in fall rather than as a food source in spring for their offspring. Hence, adult *A*. *lucorum* may be unable to forecast changes in phenology and food quality of fall-selected host plants [48,49]. Female oviposition preference and offspring performance are often weakly linked [50]. Contrary to (perennial) woody plants, insects may have great difficulty to assess the quality of (annual) herbaceous plants, which usually changes drastically over time [50,51]. During host switching, *A*. *lucorum* adults exhibit a strong preference for flowering host plants [52], with adult host plant choice related to nymphal performance during spring, summer and fall seasons [53,54]. As host plant choice and nymphal performance are poorly related between winter and spring, potential trade-offs of this drastic host switching await to be investigated. However, host plant choice during winter does not necessarily impede proper build-up of *A*. *lucorum* populations in spring, as newly hatched nymphs are often found feeding on other plant species in the vicinity of (dead) winter hosts during spring [32]. Such phenomenon is aided by the broad range of common plant species on which *A*. *lucorum* is found during spring season [33].

Between different seasons, the ratio of host plant use among the two leading families (i.e., Asteraceae and Fabaceae) and three plant growth forms was similar. We suspect that high *A*. *lucorum* use of Asteraceae and Fabaceae is not necessarily related to specific feeding preferences, but rather to the species richness and wide distribution of both plant families in northern China [37]. Similarly, herbaceous plants are far more speciose than woody plants in Chinese agro-ecosystems.

A total of 21 plant species (6 species belonging to Asteraceae; 3 species for each of Chenopodiaceae, Fabaceae and Rosaceae) served as year-long hosts of *A*. *lucorum*, but its population levels on a given host greatly fluctuated over time. For example, *A*. *lucorum* eggs overwinter on *Z*. *jujube* and *V*. *vinifera* [29,33,55], but population levels on these species greatly decreases in early summer (Fig. 3). Possibly, the ecological function of these host plants in supporting population growth of *A*. *lucorum* varies between seasons [34,56]. Other year-long host plants supported high population density in spring (*Vicia faba* L.), in summer (*Helianthus annuus* L., and *Impatiens balsamina L*.) or winter (*Pyrus bretschneideri* Rehd., and *Malus domestica* Borkh.). Additionally, the role of particular host plants/habitat patches on population dynamics of insect pests is greatly affected by landscape composition, (crop) management and other factors [34,56]. For instance, safflower is generally considered to be an important source of *L*. *hesperus* in cotton, but high insecticide application can change it into a sink; alfalfa also is a primary host plant, however it can divert *L*. *hesperus* adults away from nearby cotton depending upon the management of the former plant species [36,57]. Lu et al. [29] showed that cotton is the most important agricultural crop supporting mirid bugs in northern China during June. Before the adoption of Bt cotton, insecticide use turned cotton fields into a population sink. Presently, a notable reduction in insecticide use has led to cotton becoming a population source of this mirid species [29]. Hence, source and sink effects of various host plants and habitats on *A*. *lucorum* population dynamics need to be further investigated, especially at the landscape level.

Host breadth during spring and winter is relatively limited, and its spring density is low. This provides an opportunity for regional population suppression of *A*. *lucorum* [32,33]. Spring hosts, such as *Humulus scandens* (Lour.) Merr.,*Z*. *jujuba*, and *V*. *vinifera*, could be central in devising ways to prevent rapid *A*. *lucorum* population buildup in summer [33]. Also, *A*. *lucorum* population levels on some plant species (e.g., *V*. *radiata*, *R*. *communis*) were much higher than on others (Fig. 3), hinting at eventual host plant preferences. As olfaction plays an important role in *A*. *lucorum* host plant location [38,58], follow-up research could determine whether chemical communication also acts in *A*. *lucorum* choice of its preferred host species. Preferred host plants could then be used as trap crops or for incorporation in push-pull strategies [38,59,60,61].

In conclusion, our assessment of *A*. *lucorum* seasonal host plant use and host switching behavior helps elucidate the interaction between this polyphagous insect and its host plants, and improve our understanding of its meta-population dynamics in agricultural landscapes.
